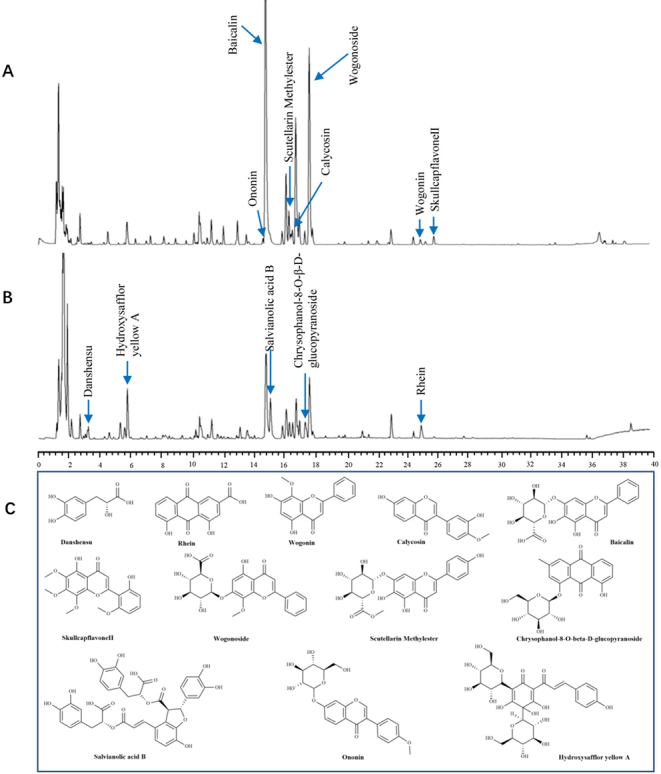# Colonic microflora and plasma metabolite-based comparative analysis of unilateral ureteral obstruction-induced chronic kidney disease after treatment with the Chinese medicine FuZhengHuaYuJiangZhuTongLuo and AST-120

**DOI:** 10.1016/j.heliyon.2024.e24987

**Published:** 2024-01-24

**Authors:** Ziwei Chen, Shaobo Wu, Li Huang, Jing Li, Xueying Li, Yu Zeng, Zejun Chen, Ming Chen

**Affiliations:** a School of Basic Medicine, Chengdu University of Traditional Chinese Medicine, Chengdu, Sichuan 610072, China; b Department of Nephrology, Hospital of Chengdu University of Traditional Chinese Medicine, Chengdu, Sichuan 610072, China; c Department of Clinical Laboratory, Hospital of Chengdu University of Traditional Chinese Medicine, Chengdu, Sichuan 610072, China; d Department of Nephrology, Affiliated Hospital of Integrated Traditional Chinese and Western Medicine, Chengdu University of Traditional Chinese Medicine, Chengdu Traditional Chinese and Western Medicine Hospital, Chengdu First People's Hospital, Chengdu, Sichuan 610072, China

**Keywords:** FuZhengHuaYuJiangZhuTongLuo recipe, AST-120, 16S rRNA sequencing, Gut microbiota, Metabolism

## Abstract

**Background:**

Many researchers have investigated the use of Chinese herbs to delay the progression of chronic kidney disease (CKD) through their effects on colonic microflora and microbiota-derived metabolites. However, whether FuZhengHuaYuJiangZhuTongLuo (FZHY) has effects that are similar to those of AST-120 on CKD needs to be elucidated.

**Methods:**

In this study, we compared the effects of FZHY and AST-120 on the colonic microbiota and plasma metabolites in the CKD rat model. We developed a unilateral ureteral obstruction (UUO)-induced CKD rat model and then administered FZHY and AST-120 to these model rats. Non-targeted metabolomic LC-MS analysis, 16S rRNA sequencing, and histopathological staining were performed on plasma, stool, and kidney tissues, respectively, and the joint correlation between biomarkers and metabolites of candidate bacteria was analyzed.

**Results:**

Our results showed that administering FZHY and AST-120 effectively ameliorated UUO-induced abnormal renal function and renal fibrosis and regulated the composition of microbiota and metabolites. Compared to the UUO model group, the *p_Firmicutes* and *o_Peptostreptococcales_Tissierellales* were increased, while 14 negative ion metabolites were upregulated and 21 were downregulated after FZHY treatment. Additionally, 40 positive ion metabolites were upregulated and 63 were downregulated. On the other hand, AST-120 treatment resulted in an increase in the levels of *g_Prevotellaceae_NK3B31_*group and *f_Prevotellaceae*, as well as 12 upregulated and 23 downregulated negative ion metabolites and 56 upregulated and 63 downregulated positive ion metabolites. Besides, FZHY increased the levels of candidate bacterial biomarkers that were found to be negatively correlated with some poisonous metabolites, such as 4-hydroxyretinoic acid, and positively correlated with beneficial metabolites, such as l-arginine. AST-120 increased the levels of candidate bacterial biomarkers that were negatively correlated with some toxic metabolites, such as glycoursodeoxycholic acid, 4-ethylphenol, and indole-3-acetic acid.

**Conclusion:**

FZHY and AST-120 effectively reduced kidney damage, in which, the recovery of some dysregulated bacteria and metabolites are probably involved. As their mechanisms of regulation were different, FZHY might play a complementary role to AST-120 in treating CKD.

## 1 Introduction

Kidney disease is a global health burden, and chronic kidney disease (CKD) affects approximately 10 % of adults worldwide [1]. In Asia, around 434.3 million adults are suffering from CKD, of which 65.6 million people are in the late stage of CKD [2]. CKD is a multiple urinary system disease, which can result in systemic inflammation and the accumulation of uremic toxins. It might eventually give rise to end-stage renal disease (ESRD), cardiovascular disease, and other complications [[3], [4], [5]]. Such complications are also the main cause of death in patients at the final stage of CKD, and they can greatly increase the cost to the social health system [5,6]. Thus, inexpensive and highly efficient therapeutic techniques for treating CKD need to be developed.

The gut microbiota is responsible for the digestion of complex carbohydrates, maintenance of intestinal epithelium, prevention of pathogen infection, regulation of the immune system and metabolites, etc. Metabolites from the intestinal flora, including various toxins, bile acids, lipids, amino acids, and organic acids, are generally metabolized and discharged in the urine, and their accumulation can cause kidney dysfunction and damage. CKD is a complex disease involving multiple factors, such as metabolism, the endocrine system, and the immune system [7]. The abundance of *Ruminococcus* and *Allobaculum* was found to be high in certain CKD animal models [[8], [9], [10], [11]]. Additionally, in the CKD animal model, an increase in the abundance of *Eggerthella lenta*, Enterobacteriaceae, and *Clostridium* spp., and a reduction in the abundance of *Bacteroides eggerthii*, *Roseburia faecis*, and *Prevotella* spp. were observed [1]. Bacterial species involved in butyrate production, indole synthesis, and mucin degradation are also related to CKD [1]. The progression or aggravation of CKD is not only related to the imbalance of the intestinal flora but also to the accumulation of metabolites that occurs as a result of the imbalance of the intestinal flora. In patients with CKD, the production of harmful metabolites (e.g., indole and p-cresol) in the colon microbiota increases, whereas the production of beneficial products (such as ketone) in the colon microbiota decreases [12]. Some studies have shown that the progression of CKD is related to gut microbiota-associated glycine-conjugated metabolites and polyamine metabolism [8]. CKD increases the production of various uremic toxins, including indole sulfate (IS), p-methyl sulfate (p-CS), trimethylamine, and trimethylamine oxide (TMAO) [13]. Damage to the kidneys of patients with CKD prevents the elimination of these metabolites, which aggravates the progress of CKD and leads to other serious complications. Regulating the gut microflora might be a promising strategy for treating CKD and its comorbidities via gut-heart and gut-brain cues [14].

The intestinal microecology in patients with CKD might be improved by administering prebiotics, probiotics, and synbiotics [15]. Traditional Chinese medicine (TCM) and the corresponding bioactive herbal components have attracted much attention as they can treat CKD by regulating the gut microbiota [16,17][18][19]. TCM improves the outcome of diseases that are not only related to the transformation of bioactive metabolites by the gut microbiota but also related to the formation of gut microbiota-induced functional lipid metabolites, amino acid metabolites, metabolic endotoxemia, and inflammatory responses [20,21]. TCM is an alternative and complementary treatment strategy. It can modulate the intestinal flora by increasing the abundance of Firmicutes and Actinobacteria and decreasing the abundance of *Corynebacterium* and *Enterococcus*. Its effects are different from those of Western medicine [22], and it can reduce the levels of indoxyl sulfate (IS), *p*-cresyl sulfate (pCS), endotoxins, and lipopolysaccharides to improve the condition of patients with CKD [23].

The traditional Chinese compound FZHY is a herbal medicine used for treating fibrosis, including renal fibrosis [24]. However, the therapeutic effects of FZHY on CKD, which is a clinical manifestation of renal fibrosis, remain unknown. FZHY in this study is an empirical prescription developed by our research team based on “treating from the spleen” for treating CKD; it can be used to treat Qi deficiency in the spleen and kidneys. FZHY contains plant and animal components to harmonize meridians [25]. We searched TCM systems in various pharmacology databases, including TCMSP, TCMID, PubChem, and BATMAN-TCM, to obtain the chemical composition of FZHY. We found that FZHY has 316 active ingredients. We visualized the “drug-ingredient-target” network to determine whether FZHY can be used for treating CKD; we found that FZHY plays an important role in treating CKD. However, the detailed chemical composition and the mechanism by which FZHY exerts anti-CKD effects are not known. The main components of FZHY, including *Astragalus membranaceus* and *Codonopsis codonopsis*, are known to control gut microbiome dysbiosis [22,26] and also reduce serum cholesterol and improve the immune function by regulating T cells to alleviate renal fibrosis [27]. AST-120 is an oral charcoal adsorbent, which is used to treat CKD [28]. It not only adsorbs uremic toxins like IS and PCS [29,30] but also regulates uremic toxin-producing gut microbiota to ameliorate renal dysfunction in patients with CKD [11,31]. However, a systematic comparison between FZHY and AST-120 has not been performed. In this study, we evaluated the therapeutic effects of FZHY and AST-120 on unilateral ureteral obstruction (UUO)-induced CKD and determined the changes in the intestinal flora and metabolites regulated by them via 16S rRNA sequencing and metabolomics. Our findings provided greater insights into the similarities and differences related to the molecular mechanisms by which FZHY and AST-120 ameliorate CKD.

## 2 Materials and methods

### 2.1 Preparation of FZHY

Using the traditional decoction water extraction method, the listed ingredients (weighed 131 g in total per dose) were dissolved in water (250 mL) at the corresponding dose (details in Table S1) for 25–30 min and boiled, followed by heating over a mild flame for 10–15 min. The sample was extracted twice with water (200 mL) and then concentrated to 2.62 g/mL and stored at 4 °C until further use. The FZHY prescription consisted of nine raw/prepared medicinal plants and animals (Table S1). All listed botanical names (details in Table S1) were validated using the Kew Medicinal Plant Names Services (http://mpns.kew.org/mpns-portal/?_ga=1.111763972.1427522246.145907734).

### 2.2 Identification of the components of FZHY by UHPLC-ESI-HRMS analysis

The FZHY solution (1.25 g/mL) was centrifuged and then the liquid supernatant was filtered using a 0.22-pm Millipore filter. The filtrate was analyzed via UHPLC-ESI-HRMS. The conditions for chromatography were as follows: a C18 column (3 mm × 100 mm, 2.6 μm; Thermo Scientific Accucore™) was used; the mobile phase was 0.1 % formic acid in water (A)-0.1 % formic acid in acetonitrile (B). Gradient elution was conducted as follows: 0–30 min, 5–50 % B; 30–35 min, 50–95 % B; 35–40 min, 99 % B. The flow rate was set at 0.3 mL/min, the column temperature was set at 30 °C, and 3 μL of the sample was loaded. Mass spectrometry conditions: both positive and negative modes were used. The spray voltage, ion source temperature, sheath gas flow rate, aux gas flow rate, and capillary temperature were 3.2 kV, 350 °C, 35 arb, 10 arb, and 320 °C. Full MS was followed by the ddMS2 mode; the mass scan range was from 10 to 1000 *m*/*z* with a resolution of 70,000 for parent ions and a resolution of 17,500 for daughter ions; the energy gradient was 20/40/60 eV.

### 2.3 Animals and the UUO model

Male Sprague-Dawley rats (n = 48, 7–8 weeks old, and weighing 240–280 g) were obtained from the Laboratory Animal Business Department, Shanghai Institute of Planned Parenthood Research (Shanghai, China). All rats were fed adaptively for one week, and then they were randomly divided into four groups: sham (n = 12), UUO (n = 12), UUO + FZHY (n = 12), and UUO + AST-120 (n = 12). In the UUO model treatment, all rats were anesthetized by intraperitoneal injection of sodium pentobarbital and fed adaptively for one week. The abdominal cavity was opened through the left abdominal incision. The left ureter was separated and double-ligated with 4–0 sutures in the middle and upper one-third, and the abdominal cavity was closed by layered suturing. The rats of the sham group underwent a similar operation, but the ureter was not ligated. The rats were used following the National Institutes of Health Guidelines for the Use of Laboratory Animals, and this study was approved by the Ethics Committee of the Hospital of Chengdu University of Traditional Chinese Medicine (approval no.: 2021DL-016). FZHY is a Chinese medicine developed by us, and AST-120 was used as a positive control as it can delay the progression of CKD. The FZHY decoction was prepared and quality-controlled, as described in another study [32]. The dose of FZHY administered to each rat was 4.92 g/kg/d [33]. The dose of AST-120 (spherical carbon adsorbent, Kureha Corporation, Japan) administered to each rat was 4 g/kg/d [34]. On the second day after operation, the chemicals were administered intragastrically once a day for 7 days, respectively. After treatment for the predetermined 7 days, seven rats were randomly selected from each group and euthanized by carbon dioxide inhalation. Stool samples, blood samples, and kidney tissues were collected for subsequent experiments.

### 2.4 Enzyme-linked Immunosorbent assay

Blood samples were obtained from rats to ascertain the presence of biochemical markers and cytokine secretion levels. Serum samples were acquired through centrifugation at a speed of 3000 rpm for a duration of 20 min. Subsequently, measurements of serum creatinine (SCR, ml058879) and blood urea nitrogen (BUN, ml730662) were performed utilizing ELISA kits (Shanghai Enzyme-linked Biotechnology Co., Ltd., Shanghai, China).

### 2.5 Western blotting

Total proteins were extracted from kidney tissue samples when treated with radioimmunoprecipitation assay (RIPA) lysis (Sangon Biotech, Shanghai, China). The concentrations of total proteins were measured by bicinchoninic acid (BCA) assay (Beyotime, Shanghai, China). The proteins were separated on SDS–polyacrylamide gel electrophoresis and transferred to an NC membrane. The NC membrane was blocked with 5 % non-fat milk and then incubated with primary antibodies against α-SMA (clone 1A4, 1:1000, A2547, Sigma-Aldrich, St. Louis, MO, USA) and fibronectin (clone 3E2, 1:1000, Sigma-Aldrich, St. Louis, MO, USA) overnight at 4 °C, respectively. Then, the membrane was washed with TBST and incubated with secondary antibodies (1:5000) at 37 °C for 2h. Finally, the proteins were monitored with enhanced chemiluminescence reagents (GE Healthcare Life Sciences, NJ, USA) and then quantitatively analyzed by ImageJ software (version 1.4.0., National Institutes of Health). GAPDH protein was used for the internal control.

### 2.6 Hematoxylin & eosin staining and Masson's trichrome staining

We evaluated the degree of renal tissue injury by hematoxylin and eosin (H&E) staining and Masson's trichrome staining. The kidney tissues were fixed in 4 % paraformaldehyde, embedded in paraffin, and sliced into thin paraffin sections (4 μm thick). Next, the sections were treated in xylene, dehydrated with graded ethanol, and stained with H&E and Masson's trichrome (Sigma-Aldrich; Merck KGaA). After staining, the sections were dehydrated with 70 % and 90 % ethanol. Six fields (magnification: 400 × ) were randomly selected and observed under an optical microscope (Olympus, Tokyo, Japan).

### 2.7 DNA extraction and 16S rRNA sequencing

The genomic DNA was obtained from rat feces and extracted using the SDS method. Then, the concentration and purity of the DNA were detected using a multi-mode microplate reader (BioTek Instruments, Inc., Winooski, VT). The genomic DNA was used as a template to amplify the V4–V5 region of the 16S rRNA gene using 16S–F and (5′-GTGCCAGCMGCCGCGG-3′) and 16S–R (5′-CCGTCAATTMTTTRAGTTT-3′) primers. Then, 20 μL of the mixture was used to conduct a PCR assay by adding the Phusion® High-Fidelity PCR Master Mix with a GC Buffer and a high-fidelity enzyme. The target bands were recovered using the GeneJET Gel Extraction Kit (Thermo Scientific). Finally, the library was constructed using the TruSeq® DNA PCR-Free Sample Preparation Kit, and 16S rRNA sequencing was performed using a NovaSeq6000 system (Novogene Bioinformatics Technology, Beijing, China).

### 2.8 Analysis of 16S rRNA

The raw data for 16S rDNA amplicon sequencing were obtained using the NovaSeq 6000 platform in the paired-end 250 bases (PE250) mode. Clean data were obtained by Trimmomatic (version 0.36), a tool used for data quality control. The Operational Taxonomic Units (OTUs) for the clean data were analyzed by UPARSE (version 7.0.1001) with a sequence similarity cutoff of 97 %. Species annotation analysis was performed using the Mothur method and the SSU rRNA database [35] of SILVA138 (http://www.arb-silva.de/). Multiple sequences were aligned using the MUSCLE [36] (Version 3.8.31, http://www.drive5.com/muscle/) software to determine the phylogenetic relationship of the representative sequences of all OTUs. The Shannon and Simpson indices were calculated using the QIIME software (version 1.9.1), and the outlier index of each group of samples was evaluated. The differences in the Shannon and Simpson indices between the groups were determined by t-tests. The vegan package of the R software (version 3.6.0) was used for conducting PCoA and plotting graphs. The significant differences in the abundance of genera were determined by the LEfSe (LDA Effect Size) analysis.

### 2.9 Metabolomics

Plasma metabolites were used for untargeted metabolomic liquid chromatography-mass spectrometry (LC-MS) analysis [37]. The spectrogram processing and database search was conducted using Compound Discoverer 3.1 to obtain the qualitative and quantitative results of metabolites. The logarithmic conversion and standardization of the data were conducted using the MetaX software [38]. Differential metabolites were screened by Partial Least Squares Discrimination Analysis (PLS-DA), and the threshold was set as follows: variable importance in projection (VIP) > 1.0, FoldChange >1.5 or FoldChange <0.667, and P < 0.05 [39]. Differential metabolites were visualized using volcano plots. The correlation between the abundance of a genus and metabolites was determined by calculating Spearman's correlation coefficient. A cutoff value of R > 0.4 and P < 0.05 was considered to indicate a significant correlation.

### 2.10 Statistical analysis

All statistical analyses were performed using GraphPad Prism 8.0 (GraphPad Software, San Diego, USA) and presented with the mean ± standard deviation (SD). One-way ANOVA (followed by the Tukey test) was performed to determine differences between groups, and the differences were considered to be statistically significant at *P* < 0.05 (*P < 0.05 and **P < 0.01).

## 3 Results

### 3.1 Chemical composition of FZHY

After re-dissolving FZHY in methyl alcohol, we conducted the UHPLC-ESI-HRMS analysis and identified 115 compounds (Fig. S1 and Table S2). Among these compounds, we identified 11 types of amino acids, 10 types of flavonoids, eight types of organic acids, seven types of alkaloids, six types of purines, five types of volatile oils, four types of glycosides, and four types of salvianolic acids. The remaining types of compounds were either less than two or classified into type Other (Table S2). According to the Traditional Chinese Medicine Systems Pharmacology Database and Analysis Platform (https://old.tcmsp-e.com/tcmsp.php), in FZHY, danshensu, hydroxysafflor yellow A, ononin, baicalin, salvianolic acid B, scutellarin methylester, calycosin, chrysophanol-8-*O*-β-d-glucopyranoside, wogonoside, wogonin, rhein, and skullcapflavone II were identified as the main chemical compounds with known therapeutic effects.

### 3.2 Administering either FZHY or AST-120 improved the kidney function of UUO model rats

**Fig. 1:**
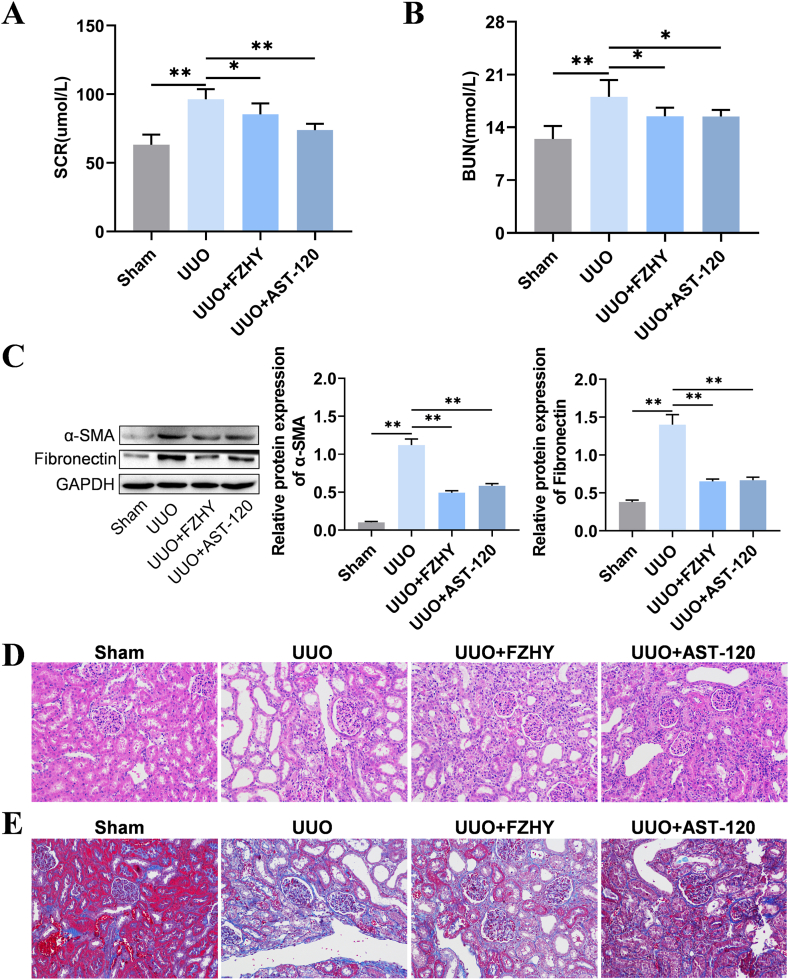
A renal injury was detected. The differences in the concentration of serum creatinine (SCR) in rats between the sham, UUO, UUO + FZHY, and UUO + AST-120 groups (**A**). The differences in the concentration of blood urea nitrogen (BUN) in rats between the sham, UUO, UUO + FZHY, and UUO + AST-120 groups (**B**). The protein expression of α-SMA and Fibronectin in renal tissue injury was detected by Western blotting in the sham, UUO, UUO + FZHY, and UUO + AST-120 groups (**C**). The renal tissue injury was detected by H&E staining (400 × magnification) in the sham, UUO, UUO + FZHY, and UUO + AST-120 groups (**D**). Renal fibrosis was assessed by Masson's Trichrome staining (400 × magnification) in the sham, UUO, UUO + FZHY, and UUO + AST-120 groups (**E**); *P < 0.05 and **P < 0.01.

To confirm whether treatment with FZHY or AST-120 can prevent the progression of CKD, we first detected the parameters of renal function and histopathological changes in the kidneys of the UUO model rats. The results of ELISA showed that the serum SCR and BUN levels were higher in the UUO model rats than in the rats of the sham group (Fig. 1A and B). The Western blotting results also demonstrated that the levels of α-SMA and Fibronectin in UUO model rats were higher than those in the sham operation group rats (Fig. 1C). The results of H&E staining and Masson's trichrome staining showed that the UUO-related kidney tissue damage manifested as renal tubular dilation, inflammatory infiltration, and renal tissue fibrosis (Fig. 1D and E). Additionally, administering FZHY and AST-120 effectively reduced the serum SCR and BUN levels, decreased the expression levels of α-SMA and Fibronectin, and decreased the structural damage to the renal tissue in the UUO model rats (Fig. 1A–E).

### 3.3 Administering FZHY or AST-120 influenced the intestinal flora of the UUO model rats

**Fig. 2:**
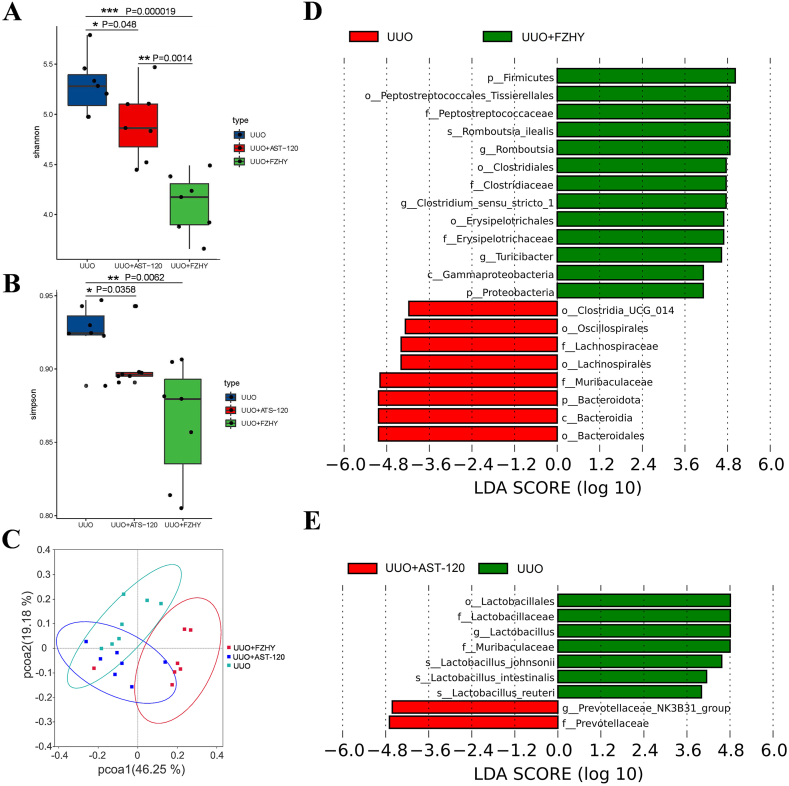
Differentially abundant microbiota under different taxonomic levels. The alpha diversity (Shannon (**A**), Simpson (**B**)) was evaluated based on the abundance of operational taxonomic units (OTU) in the UUO, UUO + FZHY, and UUO + AST-120 groups. The beta diversity was evaluated based on the PCoA plot of UniFrac distances for the UUO, UUO + FZHY, and UUO + AST-120 groups (**C**). Linear discriminant analysis (LDA) effect size (LEfSe) and cladogram analysis of the differential abundance of microbiota between the UUO and UUO + FZHY groups (**D**) and the UUO and UUO + AST-120 groups (**E**). A red bar indicates that the microbial group has a high abundance in the red group, and a green bar indicates that the microbial group has a high abundance in the green group; *P < 0.05, **P < 0.01 and ***P < 0.001; p: phylum, c: class, o: order, f: family, g: genus, and s: species. (For interpretation of the references to colour in this figure legend, the reader is referred to the Web version of this article.)

Based on the previous studies, the significant improvement of renal injury on the 7th day with FZHY and AST-120, 16S sequencing was performed on the intestinal microbiota of each group to observe whether FZHY and AST-120 regulate changes in the intestinal microbiota. To determine whether administering FZHY and AST-120 affected the composition of the microbiota, we first compared the α-diversity indices (richness and diversity) of the fecal microbiota among the UUO, UUO + FZHY, and UUO + AST-120 groups. The results showed that the Shannon and Simpson indices were significantly lower in the FZHY group than in the UUO group, and the indices were slightly higher in the AST-120 group than in the UUO group (Fig. 2A and B). We also compared the beta diversity of the gut microbiota among the UUO, UUO + FZHY, and UUO + AST-120 groups based on the weighted distance and found significant differences in the microbiota analyzed from stool samples (Fig. 2C). Additionally, we used the linear discriminant analysis (LDA) effect size (LEfSe) to identify the taxonomic biomarker bacteria that significantly contributed to the differences between the UUO and UUO + FZHY groups, and the UUO and UUO + AST-120 groups. The abundance of gut microbiota was different between the FZHY and AST treatment groups. Specifically, *p_Firmicutes*, *o_Peptostreptococcales_Tissierellales*, *f_Peptostreptococcale*, *s_Romboutsia*, *o_Clostridiales*, *f_Clostridiaceae*, *g_Clostridium_sensu_stricto_1*, *o_Erysipelotrichales*, *f_Erysipelotrichaceae*, *g_Turicibacter*, *c_Gammaproteobacteria*, and *p_Proteobacteria* were more abundant in the FZHY group than in the UUO group, whereas, *o_Clostridia_UCG_014*, *o_Oscillospirales*, *f_Lachnospiraceae*, *o_Lachnospirales*, *f_Muribaculaceae*, *p_Bacteroidota*, *c_Bacteroidia*, and *o_Bacteroidales* were more abundant in the UUO group than in the FZHY group. Between the UUO and UUO + AST-120 groups, the *g_Prevotellaceae_NK3B31_*group and *f_Prevotellaceae* were more abundant in the UUO + AST-120 group, but *o_Lactobacillales*, *f_Lactobacillaceae*, *g_Lactobacillus*, *f_Muribaculaceae*, *s_Lactobacillus_johnsonii*, and *s_Lactobacillus_reuteri* were more abundant in the UUO group (Fig. 2D and E). Among all microbial groups altered by FZHY and AST-120 in the UUO model rats, only *f_Muribaculaceae* overlapped between the treatments.

### 3.4 Administering FZHY or AST-120 improved the dysregulation of plasma metabolites in the UUO model rats

**Fig. 3:**
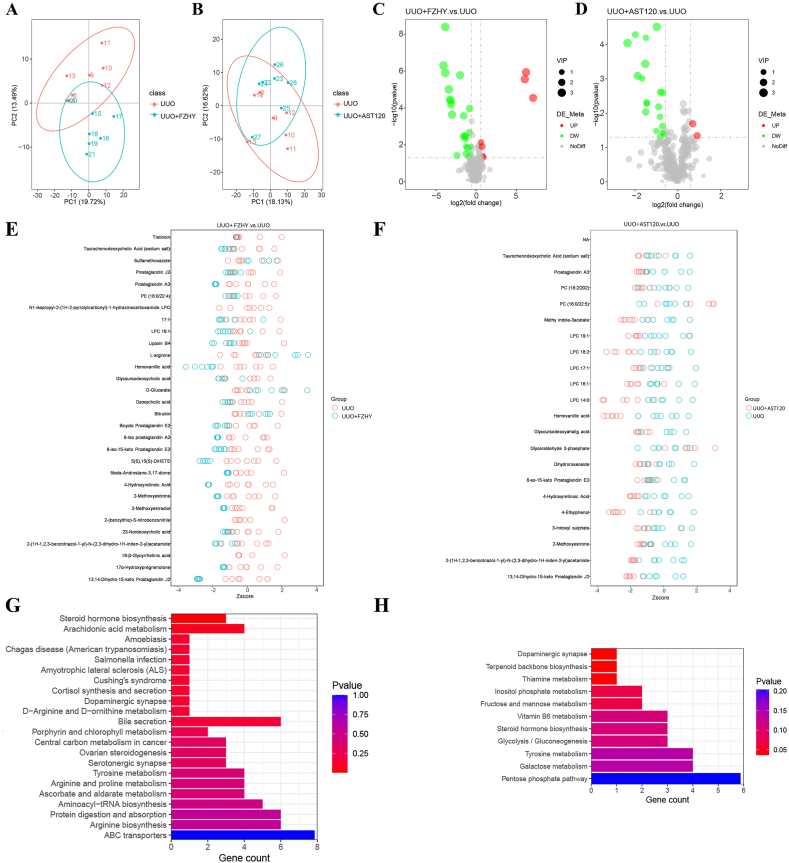
Metabolomics analysis of cationic metabolites. The PLS-DA scores were plotted to compare the global metabolite profiles in the UUO and UUO + FZHY groups (**A**) and the UUO and UUO + AST-120 groups (**B**). A volcano map was constructed for the differential genes between the UUO and UUO + FZHY groups (**C**) and the UUO and UUO + AST-120 groups (**D**). A Z-score analysis was conducted for differential metabolites between the UUO and UUO + FZHY groups (**E**) and the UUO and UUO + AST-120 groups (**F**). KEGG pathway analysis was used to identify metabolic pathways related to metabolites with rich differences between the UUO and UUO + FZHY groups (**G**) and the UUO and UUO + AST-120 groups (**H**) shown in the negative polarity pattern.

**Fig. 4:**
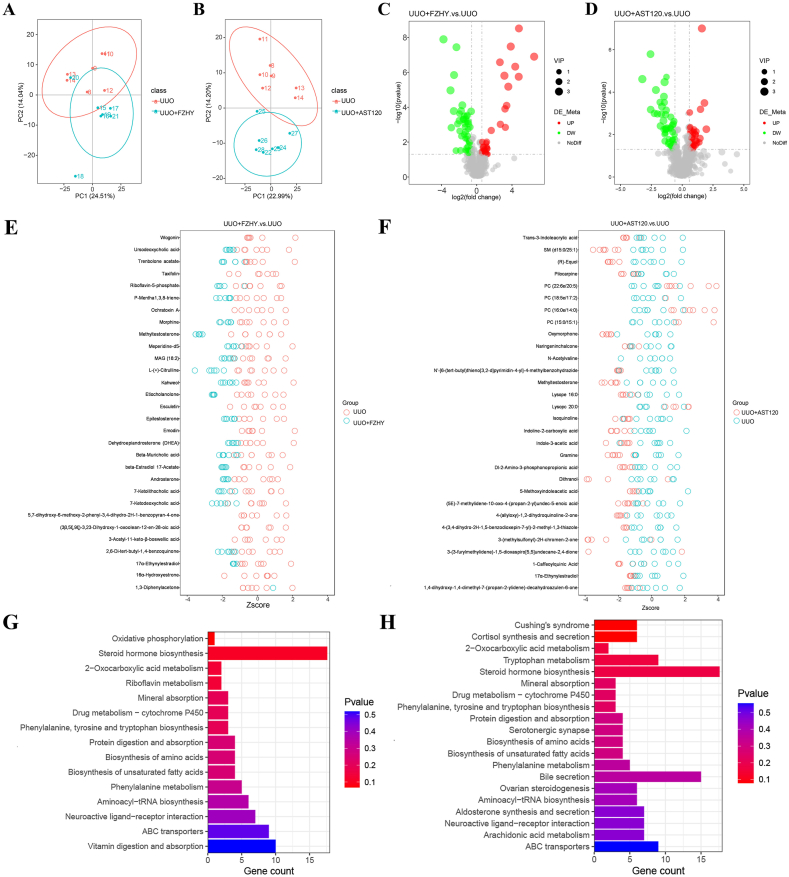
Metabolomics analysis of anionic metabolites. The PLS-DA scores were plotted to compare the global metabolite profiles in the UUO and UUO + FZHY groups (**A**) and the UUO and UUO + AST-120 groups (**B**). A volcano map was constructed for the differential genes between the UUO and UUO + FZHY groups (**C**) and the UUO and UUO + FZHY groups (**D**). A Z-score analysis was conducted for differential metabolites between the UUO and UUO + FZHY groups (**E**) and the UUO and UUO + AST-120 groups (**F**). KEGG pathway analysis was used to identify metabolic pathways related to metabolites with rich differences between the UUO and UUO + FZHY groups (G) and the UUO and UUO + AST-120 groups (H) shown in the positive polarity pattern.

To assess the roles of FZHY and AST-120 in metabolic pathways, we performed untargeted metabolomic analyses of rat plasma using an LC-MS technique. The analysis was performed using the positive ion and negative ion modes (Table S3 and Table S4). The results of the PCA showed a significant difference in plasma metabolites between the UUO and UUO + FZHY groups and the UUO and UUO + AST-120 groups (Fig. 3A and B; Fig. 4A and B). The volcano plot showed 14 upregulated and 21 downregulated negative ion metabolites in the UUO + FZHY group compared to the UUO group and 12 upregulated and 23 downregulated negative ion metabolites in the UUO + AST-120 group compared to the UUO group (Fig. 3C and D). Additionally, 40 positive ion metabolites were upregulated, and 63 metabolites were downregulated in the UUO + FZHY group compared to the UUO group, whereas 56 positive ion metabolites were upregulated and 63 metabolites were downregulated in the UUO + AST-120 group compared to the UUO group (Fig. 4C and D). The Z-scores obtained from the results of the PLS-DA showed that 30 differential cationic (Fig. 3E and F) and anionic (Fig. 4E and F) metabolites were present between the UUO + FZHY and UUO groups and the UUO + AST-120 and UUO groups. In addition, the KEGG pathway analysis of the anionic differential metabolites of UUO + FZHY group and UUO group identified a total of 22 enriched metabolic pathways, mainly enriched in Steroid hormone biosynthesis, Arachidonic acid metabolism pathways and Amoebiasis. The KEGG pathway analysis of the anionic differential metabolites of UUO + AST-120 group and UUO group revealed a total of 11 enriched metabolic pathways, primarily enriched in Thiamine metabolism, Terpenoid backbone biosynthesis, and Dopaminergic synapse pathways (Fig. 3G and H). The common anion differential metabolites between UUO + FZHY and UUO groups, and between UUO + AST-120 and UUO groups, are enriched in Steroid hormone biosynthesis, Dopaminergic synapse, and Tyrosine metabolism. The KEGG pathway analysis of the cationic differential metabolites of UUO + FZHY group and UUO group showed a total of 15 enriched metabolic pathways, mainly enriched in Oxidative phosphorylation and Steroid hormone biosynthesis pathways. The KEGG pathway analysis of the anionic differential metabolites of UUO + AST-120 group and UUO group identified a total of 20 enriched metabolic pathways, mainly enriched in Cortisol synthesis and secretion and Cushing's syndrome pathways (Fig. 4G and H). The common cation differential metabolites between UUO + FZHY and UUO groups, and between UUO + AST-120 and UUO groups, are enriched in Oxidative phosphorylation, Steroid hormone biosynthesis, 2-Oxocarboxylic acid metabolism, Riboflavin metabolism, Mineral absorption, Drug metabolism-cytochrome P450, Phenylalanine, tyrosine and tryptophan biosynthesis, Protein digestion and absorption, Biosynthesis of amino acids, Biosynthesis of unsaturated fatty acids, Phenylalanine metabolism, Aminoacyl-tRNA biosynthesis, Neuroactive ligand-receptor interaction, and ABC transporters.

### 3.5 Correlations between microbial dysregulation and metabolic product dysregulation

**Fig. 5:**
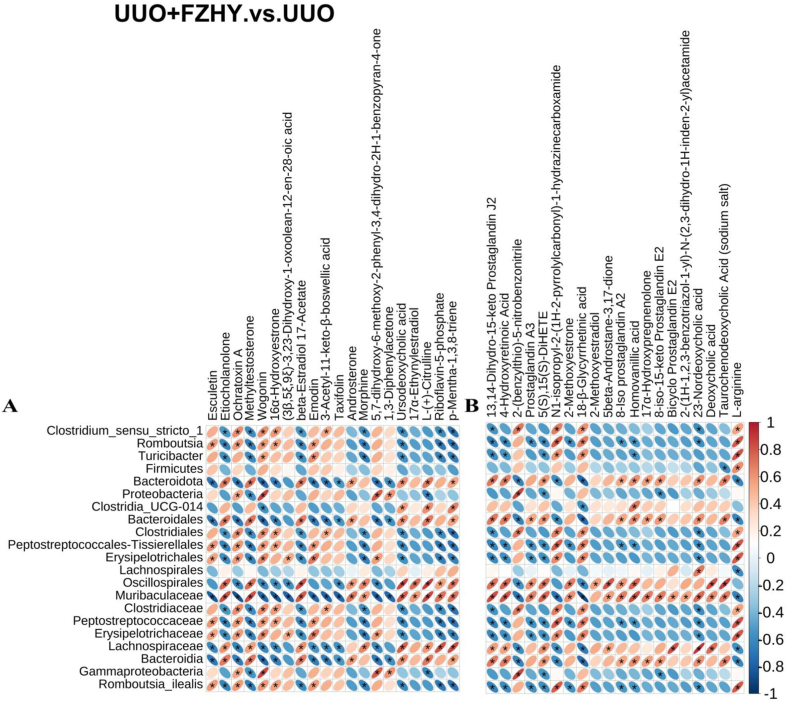
Correlation analysis between the UUO and UUO + FZHY groups. A correlation heatmap illustrating the correlation between differential microbiota and differential positive ion metabolites (**A**). A correlation heatmap illustrating the correlation between differential microbiota and differential negative ion metabolites (**B**).

**Fig. 6:**
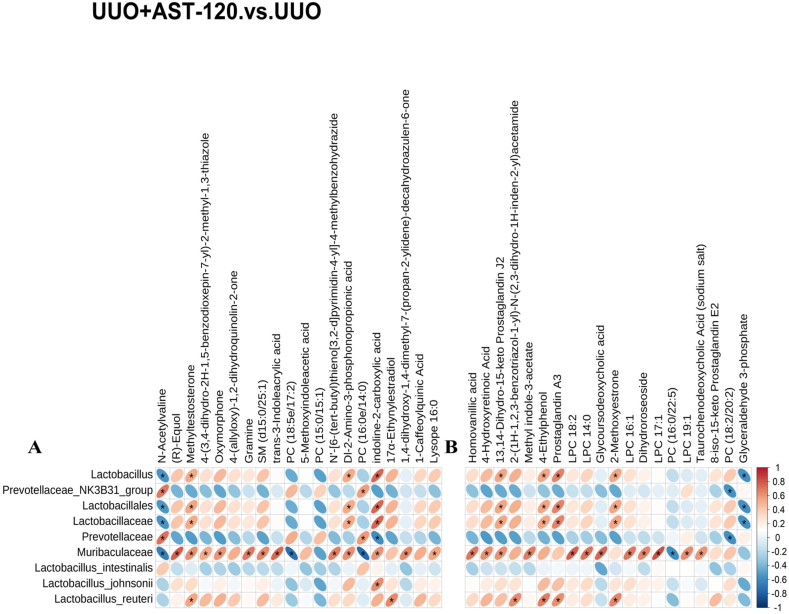
Correlation analysis between the UUO and UUO + AST-120 groups. A correlation heatmap illustrating the correlation between differential microbiota and differential positive ion metabolites (**A**). A correlation heatmap illustrating the correlation between differential microbiota and differential negative ion metabolites (**B**).

Pearson's correlation analysis was performed to examine the correlation between the 21 potential bacterial biomarkers and the top 20 positive ion metabolite markers or 20 negative ion metabolite markers between the UUO + FZHY and UUO groups. The results showed that the microbiota dysregulation improved by administering FZHY was related to amino acids, bile acids, organic compounds, etc., such as citrulline, 23-nordeoxycholic acid, and homovanillic acid (Fig. 5A and B). Pearson's correlation analysis was also performed to evaluate the correlation between the nine potential bacterial biomarkers and the top 20 positive ion metabolite markers or 20 negative ion metabolite markers between the UUO + AST-120 and UUO groups (Fig. 6A and B). The results showed that the alteration in microbiota induced by AST-120 treatment was associated with bile acids, organic acids, phenols, etc., such as glycoursodeoxycholic acid, 3-Indoxyl sulfate, and 4-ethylphenol. The differential flora regulated by FZHY and AST-120 were associated with 10 common metabolites, including cholic acid, organic compounds, and androstane steroids, mainly 13,14-dihydro-15-keto prostaglandin J2, 4-hydroxyretinoic acid, prostaglandin A3, 2-methoxyestrone, homovanillic acid, 8-iso-15-keto prostaglandin E2,2-(1H-1,2,3-benzotriazol-1-yl)-N-(2,3-dihydro-1H-inden-2-yl) acetamide, taurochenodeoxycholic acid (sodium salt), methyltestosterone, and 17α-ethynylestradiol. Between the FZHY + UUO and UUO groups, we found that these metabolites were significantly positively correlated with *f_Muribaculaceae*, but between the AST-120+UUO and UUO groups, only five types of metabolites (homovanillic acid, 4-hydroxyretinoic acid, 13,14-dihydro-15-keto prostaglandin J2, taurochenodeoxycholic acid (sodium salt), and methyltestosterone) showed a significant positive correlation with *f_Muribaculaceae*.

## 4 Discussion

A study investigated gut microbiota-related clinical outcomes in CKD and compared the effectiveness of the treatment between the TCM Jian-Pi-Yi-Shen (JPYS) decoction and kidney-targeted drug piperazine ferulate. The results indicated a better treatment outcome with TCM [22]. The effective ingredients of TCM can enter the colon and regulate the composition of the intestinal flora and its metabolites in patients with CKD. In this study, treatment with FZHY promoted the recovery of renal function and resistance of renal fibrosis in UUO rats, which was similar to the effectiveness of AST-120, although the responses of the intestinal flora and plasma metabolites to these two drugs were considerably different.

CKD is often accompanied by mild inflammation, disturbance in the intestinal flora, and dysfunction of the intestinal barrier [40]. Some studies have shown that treatment with AST-120 reversed dysbiosis of the gut microbiota in patients with CKD [11]. In this study, we showed a lower abundance of the genus *Lactobacillus* and other classification levels of lactic acid bacteria but a higher abundance of the *g_Prevotellaceae_ NK3B31*_group and *f_Prevotellaceae* in the AST-120 treatment group than that in the UUO group. A higher abundance of the genus *Lactobacillus* in the UUO rats was found to be correlated with the indicators of the severity of CKD, such as plasma tryptophan levels [41,42]. The *Prevotellaceae_UCG_001 NK3B31*_group has an anti-inflammatory effect and is negatively correlated with the pro-inflammatory factors IL-6 and TNF-α [43]. The abundance of butyrate-producing species in *f_Prevotellaceae* was significantly lower in CKD patients than that in healthy people; these species can reduce intestinal permeability and inflammation [44]. Our findings in this study match the above findings, indicating that AST-120 effectively restores the imbalanced gut microbiota.

Treatment with FZHY also affected the diversity and composition of the intestinal flora of the UUO rats. Studies have shown that a high ratio of Firmicutes/Bacteroidetes (F/B) is associated with many disease states, including CKD [45,46]. However, some studies have also found that their abundance remains unchanged in CKD [41]. In our study, administering FZHY increased the abundance of *p_Firmicute* and decreased the abundance of *p_Bacteroidota* in UUO rats. These differences in the results among studies indicate that further studies are needed to establish the significance of these microbes. The relative abundance of *o_Clostridiales* is significantly different between patients with CKD (4/5 stage) and healthy people, and this group acts as a probiotic to regulate CKD [47]. Additionally, the short-chain fatty acid-producing bacterial group *g_Clostridium_ sensu_stricto_1* was found to promote the integrity of the intestinal barrier and might help patients recover from CKD [48]. A high abundance of *o_Erysipelotrichales* has a renal protective effect and delays the progression of chronic renal failure [49]. Some studies have found that *g_Turicibacter* has an anti-inflammatory effect, and the abundances of *g_Turicibacter* and *g_Clostridium* are significantly lower in the UUO-induced CKD model. These changes are positively correlated with the reduction of tryptophan, lysine, and propionic acid, which promotes the occurrence of tubulointerstitial fibrosis in the CKD model [42,50]. The abundances of *c_Gammaproteobacteria*, and *p_Proteobacteria* are higher in the CKD model [51]. However, few studies have investigated the role of *s_Romboutsia* and *o_Oscillospirales* in CKD. Therefore, further studies are needed to find conclusive evidence.

An imbalance of the intestinal flora in rats with CKD is accompanied by changes in plasma metabolites. In our study, the results of the LC-MS analysis of kidney and plasma showed that treatment with FZHY reduced the levels of 4-hydroxyretinoic acid, 5(S),15(S)-DiHETE, 23-nordeoxycholic acid, deoxycholic acid, ursodeoxycholic acid, taurochenodeoxycholic acid (sodium salt), and citrulline and increased l-arginine levels, and these compounds were associated with the metabolism of prenol lipids, phenols, fatty acyls, bile acids, and amino acids. The relative content of hydroxyretinoic acid, which is used as a marker of chronic nephritis, is significantly increased in chronic nephritis [52]. The plasma concentration of homovanillic acid is closely related to the renal clearance rate [53]. The compound 5(S),15(S)-DiHETE is an inflammation marker derived from leukocytes, and its increase in patients with CKD might be positively correlated with inflammation [54]. Bile acids, the byproducts of intestinal bacteria, greatly accumulate in the serum of CKD patients and animals [55]. Administering FZHY can reduce the levels of 23-nordeoxycholic acid, deoxycholic acid, ursodeoxycholic acid, and taurochenodeoxycholic acid (sodium salt), which can help repair the damage caused by CKD. These results were confirmed in other studies [56]. Some studies have shown that plasma citrulline levels are relatively higher in patients with initial CKD [57]. Also, l-arginine can reduce the risk of CKD by increasing the synthesis of NO and urea, and thus, promote detoxification and the treatment of CKD [58,59]. These findings indicate that FZHY can reduce harmful metabolites in UUO rats and facilitate the treatment of CKD. The results of the correlation analysis showed that the abundance of *p_Firmicutes*, *o_Clostridiales*, *g_Clostridium_sensu_stricto_1*, *o_Erysipelotrichales*, and *g_Turicibacter* was negatively correlated with the above-mentioned five bile acids, 5(S),15(S)-DiHETE, and citrulline in FZHY-treated UUO rats, but their abundance was positively associated with l-arginine. The abundance of *p_Bacteroidota* showed the opposite pattern. Treatment with AST-120 also reduced the levels of homovanillic acid, 4-hydroxyretinoic acid, and taurochenodeoxycholic acid (sodium salt), and its effects were similar to those of FZHY. Additionally, administering AST-120 decreased the levels of glycoursodeoxycholic acid, 4-ethylphenol, methyl indole-3-acetate, 3-indoxyl sulfate, indoline-2-carboxylic acid, 5-methoxyindoleacetic acid, and indole-3-acetic acid, which are involved in the metabolism of bile acids and indoles and their derivatives. The abnormal metabolism of bile acids and toxins can aggravate CKD, as shown in other studies [55,60], including a study that examined the effects of AST-120 on CKD [61]. The results of the corresponding correlation analysis also showed that the abundance of *Lactobacillus* was positively corrected with toxins such as 4-ethylphenol and indoline-2-carboxylic acid; however, the *g_Prevotellaceae_NK3B31_* group and *f_Prevotellaceae* had no correlation with toxins and bile acids. Additionally, both FZHY and AST-120 decreased the abundance of Muribaculaceae, which plays an important role in the degradation of complex carbohydrates. In this study, it was found to be positively correlated with harmful metabolites, such as ursodeoxycholic acid, citrulline, 4-hydroxyretinoic acid, homovanillic acid, methyl indole-3-acetate, and indoline-2-carboxylic acid. Therefore, it is a potential CKD-regulating bacterial group. Moreover, from KEGG pathway analysis, we found that compared with UUO group, the differential metabolites in UUO + AST-120 group and UUO + FZHY group were enriched in steroid hormone biosynthesis and tyrosine, phenylalanine and other amino acids metabolism pathways. Studies have shown that steroids include vitamin D, and vitamin D is deficient and insufficient in patients with CKD [62]. Vitamin D can protect kidney, inhibit inflammatory process, downregulate renin-angiotensin system, prevent epithelial to mesenchymal transition and reduce parathyroid hormone [63]. Uremic retention solutes, such as IS and pCS, accumulate in CKD patients and are linked to the progression of CKD [64]. IS and pCS are sulfate conjugates of indole and p-cresol, respectively, which are produced by colon microorganisms from tryptophan and phenylalanine/tyrosine [65]. The metabolism of phenylalanine and tyrosine is significantly increased in CKD, and the enhanced metabolism of these amino acids contributes to the production of p-cresol. P-cresol is a precursor of uremic toxin that is related to the progression of CKD and can damage renal function [66]. Overall, these results suggested that FZHY and AST-120 may reverse renal injury by restoring their specific metabolites, and this function might depend on the intestinal microbiota. However, the mechanism by which FZHY and AST-120 regulate gut microbes and seral metabolite is different. AST-120 acts as a charcoal adsorbent that can directly clear uremic toxins in the kidney, such as IS, and causes secondary renoprotective effects, including rebalancing the flora and metabolism. In contrast, FZHY contains many active monomers, which not only absorb harmful metabolites from the intestine and inhibit the production of harmful metabolites but also alleviate tubulointerstitial fibrosis [67].

To determine the association between the main chemical components that were relatively abundant (labeled with an asterisk (*) in Table S2; including hydroxysafflor yellow A, baicalin, salvianolic acid B, scutellarin methylester, and wogonoside) in FZHY and the related gut microbiota. We reviewed the literature and found evidence to support our conclusions. Hydroxysafflor yellow A increased the relative abundances of the genus *Romboutsia* and decreased the F/B ratio [68]. When the intestinal flora is disturbed due to diseases, baicalin can increase the beneficial bacterial phylum *p_Firmicutes*, or restore the increased level of phylum *p_Firmicutes*, but decrease the increase in *p_Proteobacteria*, which may mitigate the disorder and restore the normal state [69,70]. Salvianolic acid B can increase the relative abundance of *p_Firmicutes*, but decrease the relative abundance of *p_Proteobacteria* [71]. However, we did not find any study on the association between scutellarin methylester (or wogonoside) and microbiota. We also did not find any published study to show the associations between the main components of FZHY and *f_Muribaculaceae*, which is influenced by both FZHY and AST-120. Regarding the effects of each of the above-mentioned main components of FZHY on kidney diseases, we found the following: baicalin can protect the kidneys from injury by suppressing NF-κB-mediated inflammation [72,73], while hydroxysafflor yellow A exerts anti-fibrosis effects by inhibiting TGF-β1-mediated epithelial-to-mesenchymal transition (EMT) in the kidneys [74]. Similarly, salvianolic acid B affects TGF-β1 signaling and EMT or Nrf2 signaling in kidney fibrosis or ischemia/reperfusion injury [75,76]. The efficacy of scutellarin methylester and wogonoside in kidney disease is not fully evaluated. However, the deglycosylated form of wogonoside, mogonin, which is a key monomer in FZHY, has a renoprotective effect [77,78]. These findings suggested that FZHY has many active components that protect the kidneys by affecting the intestinal flora. However, the relationships among the monomers in FZHY, the intestinal flora, and the anti-CKD effects are complicated and need further investigation.

The findings of few studies on the effects of each active component mentioned above on the seral metabolites in CKD, or the almost unrelated results between other studies and this study concerning seral metabolites, suggested that the anti-CKD effects of FZHY probably depend on multiple factors instead of a single component [79,80]. The findings of other studies and our results in this study indicated that most of the main active molecules in FZHY strongly influence the progression of CKD and also affect the disordered intestinal flora. However, each main active component might have different or even opposite roles when used alone or along with other components.

## 5 Conclusion

To summarize, in this study, we found that FZHY and AST-120 ameliorate renal injury and renal fibrosis. Although they cause changes in the intestinal microbiota and plasma metabolites, their mechanisms of action are different, as shown by the distinct microbiota and metabolite profiles. Our findings indicated that FZHY might serve as a complementary therapeutic agent for CKD patients treated with AST-120. However, further studies are needed to determine the anti-renal fibrosis effect of the main active components of FZHY.

## Funding

This study was supported by 10.13039/501100001809National Natural Science Foundation of China (Nos.: 81,973,673 and 82,274,487 to Ming Chen) and Science and Technology Foundation of Sichuan Province (No.: 2021YFS0034 to Ming Chen).

## Data availability statement

The data that support the findings of this study have been deposited into CNGB Sequence Archive (10.13039/100017120CNSA) [81] of China National GeneBank DataBase (CNGBdb) [25] with accession number CNP0002488 and CNP0002493.

## CRediT authorship contribution statement

**Ziwei Chen:** Writing – original draft, Software, Methodology, Formal analysis, Data curation. **Shaobo Wu:** Writing – original draft, Software, Methodology, Formal analysis, Data curation. **Li Huang:** Visualization, Software, Resources, Methodology, Formal analysis, Data curation. **Jing Li:** Visualization, Software, Resources, Methodology, Formal analysis, Data curation. **Xueying Li:** Visualization, Software, Resources, Methodology, Data curation. **Yu Zeng:** Visualization, Software, Resources, Methodology, Formal analysis. **Zejun Chen:** Writing – review & editing, Visualization, Software, Resources, Methodology, Formal analysis. **Ming Chen:** Writing – review & editing, Validation, Supervision, Software, Project administration, Investigation, Funding acquisition, Conceptualization.

## Declaration of competing interest

The authors declare that they have no known competing financial interests or personal relationships that could have appeared to influence the work reported in this paper.

## Figures and Tables

**figs1:**