# Supplementary material for: Colonic microflora and plasma metabolite-based comparative analysis of unilateral ureteral obstruction-induced chronic kidney disease after treatment with the Chinese medicine FuZhengHuaYuJiangZhuTongLuo and AST-120
Source: Heliyon. 2024 Jan 24;10(3):e24987. doi: 10.1016/j.heliyon.2024.e24987 (PMC10850519; doi:10.1016/j.heliyon.2024.e24987)
Supplement: Multimedia component 1 [file mmc1.docx]

**Table S1.** The composition of FZHY (including raw/prepared medicinal plants and animals)

| **Herbal name (Chinese name)** | **Herbal Ingredients of each dose (g)** |
| --- | --- |
| *Astragali Radix* | 40 |
| *Radix Rehmanniae Praeparata* | 20 |
| *Radix Glycyrrhizae* (Raw) | 6 |
| *Radix Salviae Miltiorrhizae* | 15 |
| *Carthami Flos* | 20 |
| *Eupolyphaga/Steleophaga* | 10 |
| Wine-processed *Scutellariae Radix* | 10 |
| Wine-processed *Rhubarb* | 5 |
| Wine-processed *Hirudo* | 5 |
