# Supplementary material for: Colonic microflora and plasma metabolite-based comparative analysis of unilateral ureteral obstruction-induced chronic kidney disease after treatment with the Chinese medicine FuZhengHuaYuJiangZhuTongLuo and AST-120
Source: Heliyon. 2024 Jan 24;10(3):e24987. doi: 10.1016/j.heliyon.2024.e24987 (PMC10850519; doi:10.1016/j.heliyon.2024.e24987)
Supplement: Multimedia component 2 [file mmc2.docx]

| **Name** | **Formula** | **Calculated Molecular weight** | ***m/z*** | **Retention time** | **Corresponding Chinese medicine** |
| --- | --- | --- | --- | --- | --- |
| Danshensu | C_9_H_10_O_5_ | 198.05249 | 197.04514 | 3.19 | *Radix Salviae Miltiorrhizae* |
| Hydroxysafflor yellow A* | C_27_H_32_O_16_ | 612.16806 | 611.15959 | 5.80 | *Carthami Flos* |
| Ononin | C_22_H_22_O_9_ | 476.13179 | 477.14016 | 14.70 | *Astragali Radix* |
| Baicalin* | C_21_H_18_O_11_ | 446.08390 | 447.09116 | 14.87 | *Scutellariae Radix* |
| Salvianolic acid B* | C_36_H_30_O_16_ | 718.15555 | 717.14718 | 15.15 | *Radix Salviae Miltiorrhizae* |
| Scutellarin Methyl ester* | C_22_H_20_O_12_ | 476.09453 | 477.10168 | 16.41 | *Scutellariae Radix* |
| Calycosin | C_16_H_12_O_5_ | 284.06743 | 285.07556 | 16.52 | *Astragali Radix* |
| Chrysophanol-8-O-β-D-glucopyranoside | C_21_H_20_O_9_ | 416.11054 | 415.10324 | 17.52 | *Rhubarb* |
| Wogonoside* | C_22_H_20_O_11_ | 460.09951 | 461.10669 | 17.72 | *Scutellariae Radix* |
| Wogonin | C_16_ H_12_O_5_ | 284.06734 | 285.07532 | 25.01 | *Scutellariae Radix* |
| Rhein | C_15_H_8_O_6_ | 284.03217 | 283.02490 | 25.04 | *Rhubarb* |
| Skullcapflavone II | C_19_H_18_O_8_ | 374.09952 | 375.10675 | 25.88 | *Scutellariae Radix* |

**Table S2.** The main chemical compounds present in FZHY

* Relative abundant component
