# Supplementary material for: Colonic microflora and plasma metabolite-based comparative analysis of unilateral ureteral obstruction-induced chronic kidney disease after treatment with the Chinese medicine FuZhengHuaYuJiangZhuTongLuo and AST-120
Source: Heliyon. 2024 Jan 24;10(3):e24987. doi: 10.1016/j.heliyon.2024.e24987 (PMC10850519; doi:10.1016/j.heliyon.2024.e24987)
Supplement: Multimedia component 3 [file mmc3.pptx]

## Slide 1
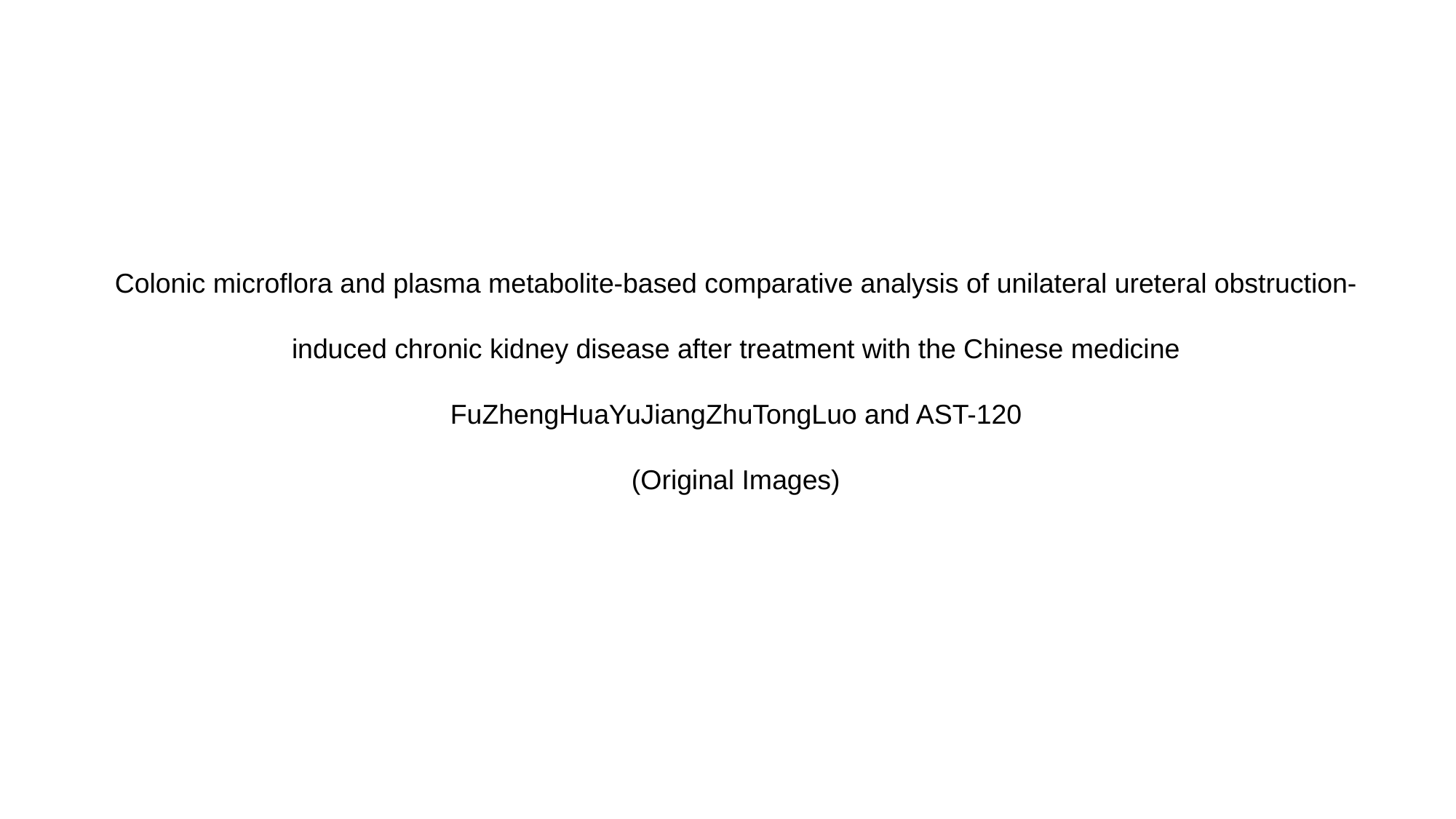

Colonic microflora and plasma metabolite-based comparative analysis of unilateral ureteral obstruction-induced chronic kidney disease after treatment with the Chinese medicine FuZhengHuaYuJiangZhuTongLuo and AST-120
(Original Images)

## Slide 2
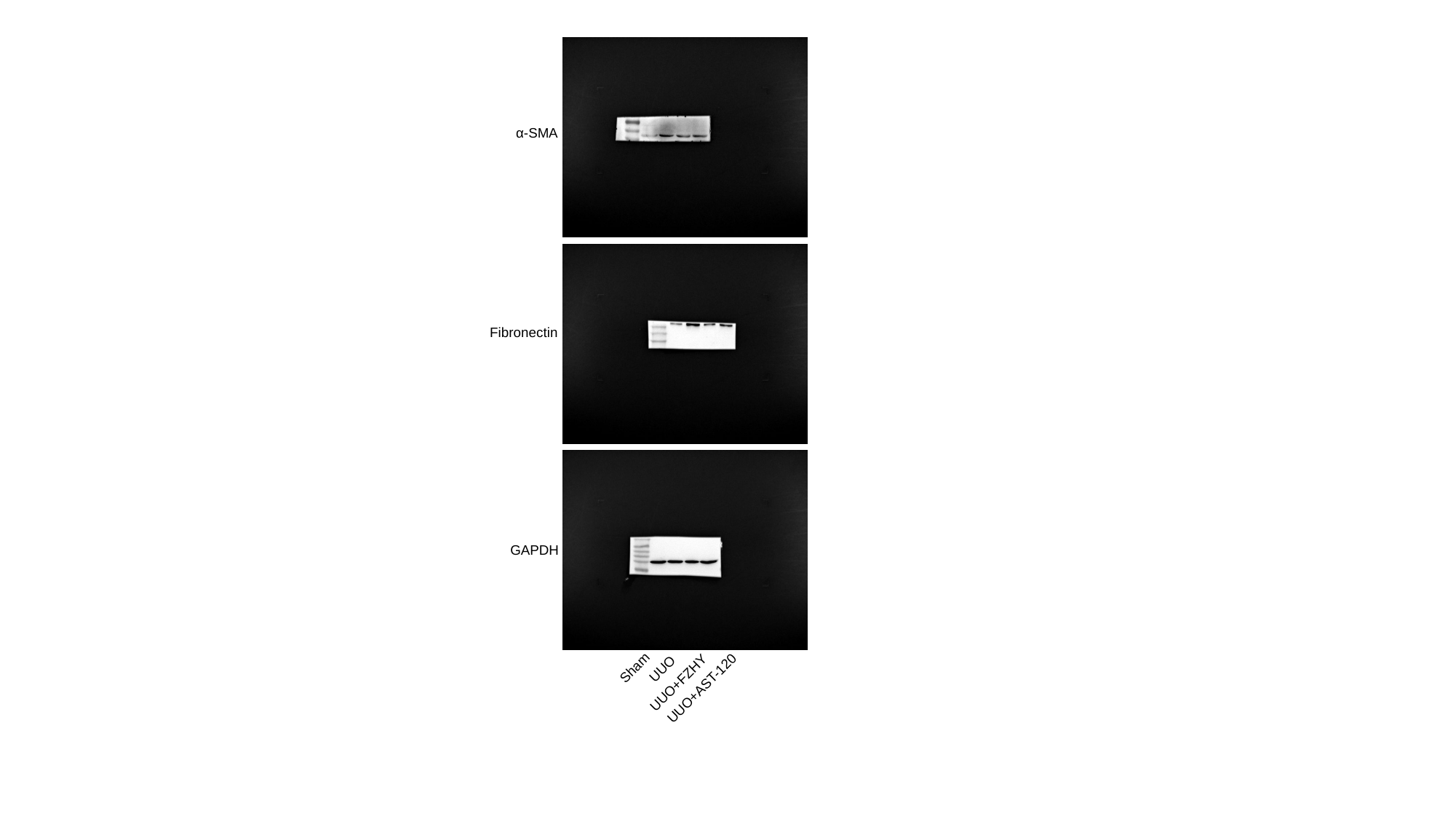

α-SMA
Fibronectin
GAPDH
Sham
UUO
UUO+FZHY
UUO+AST-120
